# Facial Pain Expression Recognition in Real-Time Videos

**DOI:** 10.1155/2018/7961427

**Published:** 2018-10-30

**Authors:** Pranti Dutta, Nachamai M

**Affiliations:** ^1^Department of Computer Science, Christ University, Bengaluru 560029, India; ^2^Siemens India, Bengaluru 560100, India

## Abstract

Recognition of pain in patients who are incapable of expressing themselves allows for several possibilities of improved diagnosis and treatment. Despite the advancements that have already been made in this field, research is still lacking with respect to the detection of pain in live videos, especially under unfavourable conditions. To address this gap in existing research, the current study proposed a hybrid model that allowed for efficient pain recognition. The hybrid, which consisted of a combination of the Constrained Local Model (CLM), Active Appearance Model (AAM), and Patch-Based Model, was applied in conjunction with image algebra. This contributed to a system that enabled the successful detection of pain from a live stream, even with poor lighting and a low-resolution recording device. The final process and output allowed for memory for storage that was reduced up to 40%–55% and an improved processing time of 20%–25%. The experimental system met with success and was able to detect pain for the 22 analysed videos with an accuracy of 55.75%–100.00%. To increase the fidelity of the proposed technique, the hybrid model was tested on UNBC‐McMaster Shoulder Pain Database as well.

## 1. Introduction

The International Association for the Study of Pain (IASP) defines pain as an “unpleasant sensory and emotional experience associated with actual or potential tissue damage, or described in terms of such damage” [1]. As this definition displays, the detection of pain in the body is a useful indicator of problems or complications, such as injury. While the incidence of pain can be defined clearly, the feeling and severity that each individual undergoes is a subjective experience, differing from person to person [2]. This subjectivity introduces difficulties in diagnosis and treatment of patients. The assessment of pain is a useful clinical criterion for the detection of underlying conditions, some of which are underrecognised. Patients suffering from neurological diseases such as Parkinson's disease, autoimmune disorders such as rheumatoid arthritis, and other conditions such as cancers and endometriosis all experience pain symptoms [3–6]. The ability to accurately recognise the occurrence of pain in a clinical setting is therefore crucial to help patients.

Currently, the assessment of pain is carried out by means of self-report, external observations, or physiological tests [7]. Among these three methods, self-report, where the patients describe their own symptoms and experience, is the most preferred. External observation allows for error and is time-consuming, while physiological tests require the use and possession of sophisticated equipment that may not always be available [8]. However, even the favoured method of self-reporting suffers from several drawbacks. Not all patients are able to express their experience of pain, such as patients in a coma or neonates [9]. The subjectivity of the event also creates another barrier for the accurate perception and diagnosis of pain [10]. The various shortcomings that exist in the contemporary system have contributed to the investigation of computer-based automated recognition of pained expressions.

Facial expressions were previously considered as a useful checkpoint for the identification of different emotions, including pain. The Facial Action Coding System (FACS), introduced by Ekman, was commonly applied for the better understanding of pain using facial features [11]. The detection of pain involved the analysis of permanent and transient facial features [12]. Conventional approaches focussed on the analysis of still images, but this limited their application to clinical settings [13]. Facial expressions encountered during the course of everyday life and in a clinical setting were not limited to a single, still image, but were composed of multiple images that were in motion. Additionally, more information was gleaned from several related images that were observed in succession than from a solitary photo [14]. For these reasons, research is now shifting away from the dependence on static imagery, and more weightage is being given to the continuous and automatic monitoring of dynamic videos. The ability to detect pain from real-time video streams presented several advantages, primarily in the reduction of time needed for assessment. It also reduced the equipment required for diagnosis. Computer-based detections and software limited the influence of subjectivity on diagnosis, and the information gained from such studies has the potential to add to our knowledge and experience about pain [15].

## 2. Review of the Literature

The efficiency and advantages of automated monitoring of facial expressions, especially pain, have been investigated in several reports. The present study utilised eigenvectors as part of the adopted hybrid model. The researcher presented the concept of a neural network to help in the easier identification of a pained expression. Monwar and Rezaei introduced eigenimages to form a template used for the detection and recognition of pain [16]. Monwar et al. reviewed the use of these eigenimages as an appropriate method to detect pain [17]. Faces were identified from video input using skin colour data, and the eigenimages were used to detect pain. Overall, it was concluded that although the system produced satisfactory results, there was a need to focus on real-time video sequences.

The use of a Patch-Based Model was advocated as early as 2006 by Hegerath et al. [18], who discussed the use of this model to recognise and identify objects, specifically faces. According to their research, faces could be divided into a number of patches that were used as the sole classifiers for the determined object. The use of Gaussian densities, along with absolute and relative patch positions, was demonstrated to create an approach that successfully identified the object in question. Hu et al. discussed the recognition of faces in video input using various patches [19] and advocated the input of data from video, followed by the individual extraction of the faces from the video feed. The facial patches were then recombined and reconstructed to allow for accurate recognition. The study reported above 80% accuracy as a result of their experiment.

Jiang et al. defined patches as fundamental units that could be used to identify an object, including a face, and included the combination of a patch-based model and principal component analysis. They opined that patches were more useful in the recognition and identification of an object than pixels [20]. The authors discussed the use of automated pain detection using machines that had been trained using facial action units. In their study, machines differentiated between real pain induced by cold sensation and artificial pained expressions with an accuracy rate of close to 75%, indicating the potential of this technique. The concept of Euclidean distances, which was applied to the present study [21]. A machine learning approach for the successful identification of pain was advocated by the authors [13], who discussed the use of machines to detect pain in individuals wherein pain was inflicted through thermal stimulation.

The importance of using nonhuman observers to detect pain was established by Ashraf et al., who used Active Appearance Models to help detect pained expressions on injured respondents. The authors used Procrustes analysis to align facial features [22]. This analysis produced better results than merely using AAM for analysing the shape features. They also introduced a relevance vector regression to predict pain. Gholami et al. [23] asserted that the use of computer-based pain recognition showed similar accuracy and adequacy when compared to human assessment of pain, highlighting its importance when used in a clinical setting. They conducted an additional study that discussed the effectiveness of computer-based pain recognition and focussed on the application of relevance vector machine learning to successfully provide a qualitative and quantitative assessment of pain in neonates. Their findings provided venues for clinical pain relief and treatment, not just of neonates, but also for other patients who were unable to vocally express pain [9].

Lucey et al. used data collected from the videos of individuals with shoulder injuries and extracted data from individual frames [24]. The data were analysed using an Active Appearance Model (AAM) which covered the extraction of the shape, appearance, and information related to the transient and permanent features. While some positive identification was made, complications arose due to movement of the head during analysis. Lucey et al. discussed the importance of pain monitoring in patients but emphasized that human monitoring demanded time and resources that were not available [8]. They proposed using automated recognition systems that depended on facial action units and released a significant fraction of an existing database related to pained faces and expressions. Information from this database was used by Kaltwang et al., who provided suggestions for the first entirely automated system designed to detect pained facial expressions [25]. Werner et al. simplified automated pain detection and introduced a new system of training in which a machine uses comparative learning for pain detection [26].

Werner et al. developed a database that served as the reference for expressions of pain [27]. In the BioVid Heat Pain Database, information from the videos and images of 90 participants were collected, all of whom were experiencing pain. The database included references for facial expressions and corresponding head positions associated with pain. In 2014, Werner et al. [7] built on their work, recommending improvements such as eliminating noise from the video background. They still suffered drawbacks, especially in those individuals wherein there was less expression of pain, and this prompted them to conduct a study in 2017. In this case, the researchers used facial descriptors to accurately measure and assess pain on the respondents' faces. They found that this alternate system was much more effective than the previous efforts that had been made. They proposed suggestions for earlier shortcomings, such as the use of biomedical factors to overcome limited expressions of pain. Another data set was created by Zhu, which specifically targeted the identification of pain from pained faces in video input [28]. This study organised the pained expressions into four categories, based on the severity of pain that was being experienced and used a combination of the probabilistic latent semantic analysis model and visual words to detect and analyse pain. Zhu used action units as a means of precisely defining and detecting pain.

Bartlett et al. analysed the use of computer vision (CV) and the recognition of patterns to investigate whether computers could distinguish between genuine and false expressions of pain [29]. The researchers used two sets of videos to examine the facial expressions of pain, dividing participants based on whether the pain they exhibited was genuine or false. The videos were analysed using a CV system called CERT, the Computer Expression Recognition Toolbox. The findings from their study indicated that there were advantages to using an automated system for expression recognition when compared to human observation and manual coding.

Recent developments in this field involved expansions of the patient group and improvement of the research equipment. For instance, Lo Presti and La Cascia described an innovative approach of using novel facial descriptors to detect pain [30]. Yan utilised the concept of a Constrained Local Model (CLM) to distinguish between pained and nonpained faces [31]. Krishna et al. focussed on children as patients [32]. They used the Viola–Jones algorithm with a high success rate. This algorithm involved the detection of the overall face, followed by the extraction of specific features. The same algorithm was adapted for the present study. Hasan et al. combined eigenfaces and support vector machines to process information from videos that came from a smartphone [33]. The researchers' method to create a simple, cost-effective method to detect pain was successful. Principal Component Analysis (PCA), which uses eigenimage pain detection, was used with Gabor filtering by Roy et al. to automatically detect pain [34]. In this approach, the entire face was first considered as an eigenimage, and then specific features were extracted from different locations in the face. This method was met with a high rate of success, and the use of PCA was thus incorporated into the present study. Kharghanian et al. designed a new approach targeting the continuous detection of pain in patients [9]. They extracted relevant facial features from frames of video and used suitable filters and vectors to distinguish between the images showing pain and those that did not. A kernel-based algorithm coupled with a relevance vector machine was used to detect pain [35].

The compiled information indicated that while the importance of automated facial recognition of pain has been established and some research has been carried out, there is still more work that needs to be conducted in the field of automatic pain detection, specifically in the areas of real-time video streaming and continuous assessment of pain. The present study thus adopted a hybrid model, combining AAM and PCA, which had been met with success in their previous applications, with a Patch-Based Model and CLM.

Previous research had focussed on the detection of pain from static images and had controlled the external environmental conditions to obtain the best possible results. In contrast to this, the current study used information extracted from videos and was able to produce reliable results under varying lighting conditions, including poor lighting situations and head pose variations. The system was also capable of functioning when the information was procured from live-streamed data and the researcher utilised a raw homemade data set. Other constraints that had been faced by previous researchers, including occlusions and lighting conditions, were also considered in this study, and the resultant model that was developed aimed at being able to provide a system that was able to successfully detect pain in subjects, overcoming previous limitations with satisfactory accuracy (measured by the hit rate).

Image-based algebra and training of the system were carried out as well. The incorporation of image algebra into the process was useful as it contributed towards the reduction of memory required for the storage and processing of data. This presented huge advancements in terms of the hardware and software requirements. The present study also aimed at creating a model that could identify pain, even when it was expressed along with other emotions during a live stream. Input was collected from varying devices, and a variety of formats were analysed using the proposed model.

## 3. Materials and Methods

Input was provided to the system in the form of a video, using formats such as .avi, .mp4, .wmv, and .mov. Live-streamed video obtained using a laptop web camera was also provided as input data. From the input, frames that contained a face were extracted. Following this, the extracted facial images were preprocessed, wherein they were subjected to face detection and cropping. From these images, the frame-by-frame detection and continuous tracking of specific facial feature points were carried out. Pain was detected using 3D face pose dynamics (specifically the yaw, pitch, and roll). Additionally, other indicators were taken into account to allow for greater accuracy of the detection of pain. These included scale parameters, the shift from the *x*-axis, shift from the *y*-axis, and the local binary pattern.

As input was provided in a live setting, it was necessary to overwrite the previous frames and to refresh and update the system with the arriving data. Automatic control of the buffer data that was present in the live stream was crucial for more accurate assessment of the required images. Information from the live stream was deposited and converted into a stored data set. In this data set, the image information could be stored and retrieved in the matrix form without the loss of any data as temporal information. This same image could then be recovered from the matrix when required. Using data from the stored images, the researcher classified the images associated with pain into three types: pain_face, not_pain_face, and neutral_face.

### 3.1. Preprocessing

Preprocessing of the input involved three steps. The preliminary step involved the extraction of the required frames from videos. Following this, the facial region was located within the window of the video, and the final step involved the enhancement of the quality of the image frames, which was carried out by the removal of unwanted noise from the context. This stage led to better results in the pain detection system. Frame extraction was accomplished by applying colour conversion, which allowed for the conversion of RGB to YCbCr. Face detection was carried out using the Viola–Jones method, and the face was cropped to a 64 ∗ 64 matrix. The shot detection algorithm was applied to each video frame in order to omit frames that did not possess information along with the proper face alignment [36].

**Figure 1:**
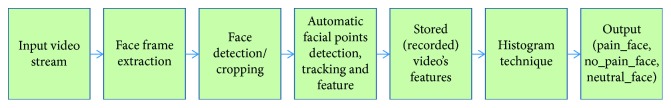
Diagrammatic representation of the system used in the study.

The flow of the system is pictorially represented in Figure 1.

### 3.2. Facial Feature Point Detection, Tracking, and Feature Extraction

Following the detection of the face in each frame, specific information known as facial feature points, facial fiducial points, or facial landmark points was extracted for further classification. The detection of facial feature points and their localisation served as the two intermediate steps in the flow. Following this, the localised points were tracked and the feature points were extracted. These steps remained extremely complex in real-time implementation owing to a moving background, variations in the illumination, poses, occlusions, over fitting, local minima, and dependency on the initial shape. To overcome these complexities, a hybrid model was structured, which was based on the concepts of AAM, CLM, and a Patch-Based Model.

The role of AAM in the hybrid model was to obtain an ideal global texture in order to overcome the problem of local minima. Statistically, AAM was defined in terms of shape based on the formula provided in the following equation:

(1) $$\begin{array}{c} s=s_{0}+\overset{k}{\underset{i=1}{\sum }}q_{i}s_{i}, \end{array}$$

where *s* is the shape of an AAM that creates a mesh structure with vertices *v*, i.e., *s*=(*a*_1_ *b*_1_, *a*_2_ *b*_2_, *a*_3_ *b*_3_,…,*a*_*v*_ *b*_*v*_)^T^and *s*_0_ is the base shape, which is summed to the linear combination of *k* shape vectors *s*_*i*_, *q*_*i*_, which are the coefficients of shape parameters. In this study, nonrigid shape vectors were used.

Following this, PCA was applied to the training shape meshes. These training meshes were normalised by Procrustes analysis before using PCA. Procrustes analysis is a preprocessing method that is used to remove deviations owing to global transformation and base shape estimation. PCA aided in the calculation of the base shape *s*_0_ (mean shape) and the covariance matrix, along with eigenvectors (*s*_*i*_), corresponding to the *k* largest eigenvalues.

AAM was defined in terms of appearance using the following equation:

(2) $$\begin{array}{c} A\left(z\right)=A_{0}\left(z\right)+\overset{1}{\underset{i=1}{\sum }}\lambda_{i}A_{i}\left(z\right), \end{array}$$

where *A*(*z*) is the appearance of an AAM within the base shape *s*_0_, *A*_0_(*z*) is the base appearance of image *Z*, which is summed to the linear combination of *l*, and the appearance images *A*_*i*_(*z*) and *λ*_*i*_ are the coefficients of appearance parameters and *z* ∈ *s*_0_.

Similar to shape, the base appearance *A*_0_(*z*) and appearance image *A*_*i*_(*z*) were also computed using PCA, with *A*_0_(*z*) set to mean image and *A*_*i*_(*z*) with *l* eigenimages corresponding to the *l* largest eigenvalues.

The increase of the real-time performance (upto 15–20 fps) and face alignment performance trailed by tracking speed was brought about by merging the CLM method with the AAM approach.

CLM consisted of two models: the shape model and the patch model. For the application of this model, a training data set of 30–50 fps peak frame rates for each facial expression was prepared. Each face had a total of 66 facial feature points annotated for it, and a shape model was built based on the features that were extracted from these feature points. The final model that was proposed in the current study had a face vector of 132 numbers for each face shape. Procrustes analysis had already been completed to align these shapes, and PCA was conducted to obtain the mean shape vector, the eigenvalues, and the eigenvectors. Thus, the shape model was developed.

**Figure 2:**
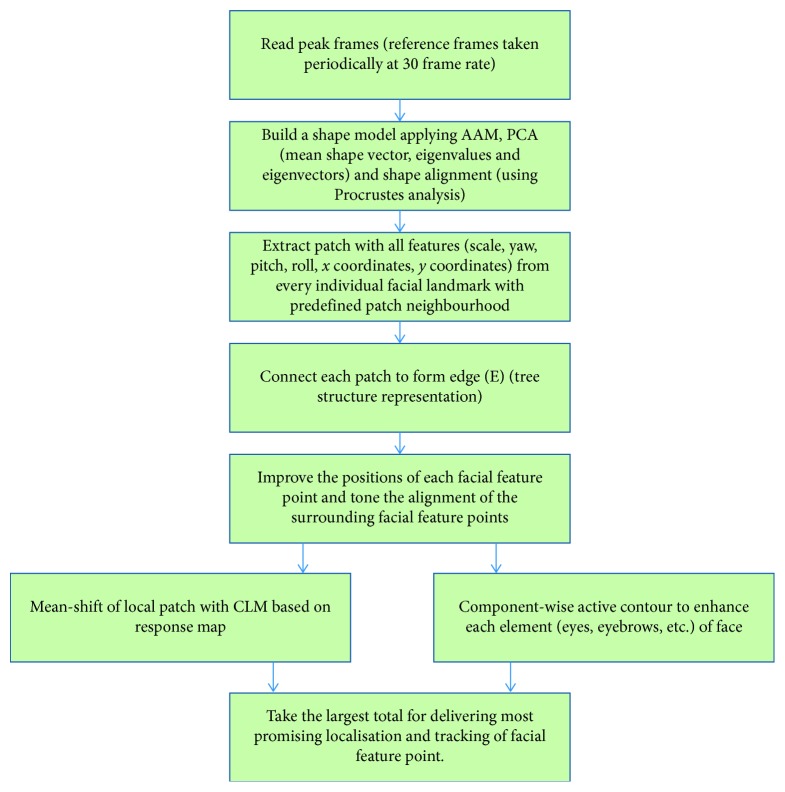
Design of the proposed hybrid model.

The patch model was developed by extracting patches from peak frames (reference frames). As every face frame had 66 facial features, 66 patches were cropped from each face frame. However, from person to person, the size and angle of rotation were different. The patch-based concept was then applied. The patch-based approach contained different parts of the same class. The implemented hybrid model approximately predicted the appearance and shape to find the root points for each patch of the face separately. From each patch, localised information was extracted to build a comparative location between the patches. Scale, yaw, pitch, roll, shift of *x* coordinates from the reference shape, and shift of *y* coordinates from the reference shape were the features extracted from each of the facial points with predefined points surrounding each extracted point. The enhancement of the positions of the individual facial feature points and toning of the relative positions of the surrounding facial feature points involved the use of a local patch mean-shift using CLM and component-wise active contour approach. A tree connectivity structure was applied to build connections between the individual units. For each visible facial point *i*, a tree was defined, as shown in the following equation:

(3) $$\begin{array}{c} T_{i}=\left(V_{i}E_{i}\right),\;i\in \left\{1,2,\ldots ,n\right\}, \end{array}$$

where *V*_*i*_ represents the vertices that represent the parts and *E*_*i*_ stands for the edges that represent the connections between these parts. A schematic representation of the hybrid model used in this study is provided in Figure 2.

Image algebra was also used for this study, which is a mathematical theory that is concerned with image transformation and image analysis. It primarily concentrates on digital images, as well as on the analysis and transformation of images by computers. The role of image algebra is to provide a rich mathematical structure that can be exploited in computer vision. The current study utilised point-based image representations and point values instead of neighbourhood pixel-based representations and coordinate values. This allowed for the incorporation of two point operations, namely, a log operation and an exponential operation. Image algebra aided in image enhancement, filtration, and examination of the required elements. The construct helped reduce memory space needed for the storage and processing of image data and allowed for information to be obtained from the input images. The combination of image algebra along with the hybrid model provided a unique potential to detect pain from the source faces.

#### 3.2.1. Logarithm Operation

The logarithm operation was a transformation operation that was used to amplify the values of dark pixels, while simultaneously compressing the values of bright pixels leading to the alleviation of contrast of the brighter region:

(4) $$\begin{array}{c} s=c\;\log\left(1+r\right), \end{array}$$

where *s*  is the  output, *c*  is  any constant (that could be changed to bring about a corresponding change in the brightness), and *r*  is the  image or frame that was being processed.

**Figure 3:**
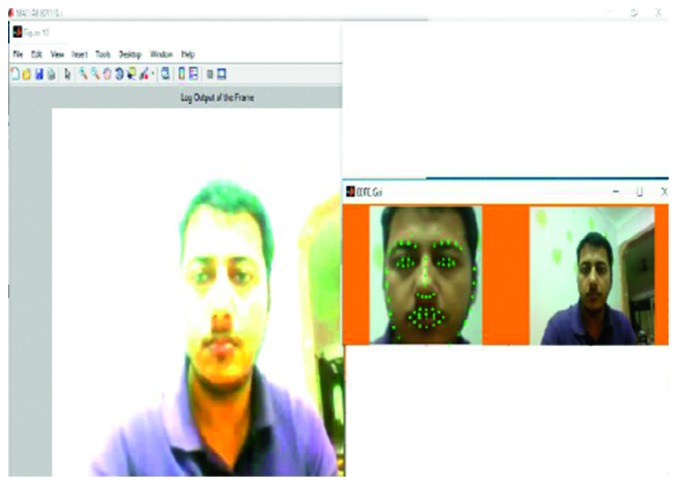
Output of logarithm operation.

The logarithm operation that was used in the study is depicted in Figure 3, with the constant *c* set at a value of 3.5. The contrast was changed according to the requirement of the situation. An increase in the constant value brought about a reduction in the contrasts of the brighter regions. In the current study, it was seen that when the value of *c* was set at 3.5, this contributed to the best results for feature extraction.

#### 3.2.2. Exponential Operation

This transformation operation was used to ameliorate the contrast of brighter regions.

(5) $$\begin{array}{c} s=c\;*\;\left(r\gamma \right), \end{array}$$

where *s*  is  the output pixel value, *r*  is  the input pixel value, and *c* and *γ* are real numbers (*c* is a factor and *γ* is the exponential power of the image or the frame).

**Figure 4:**
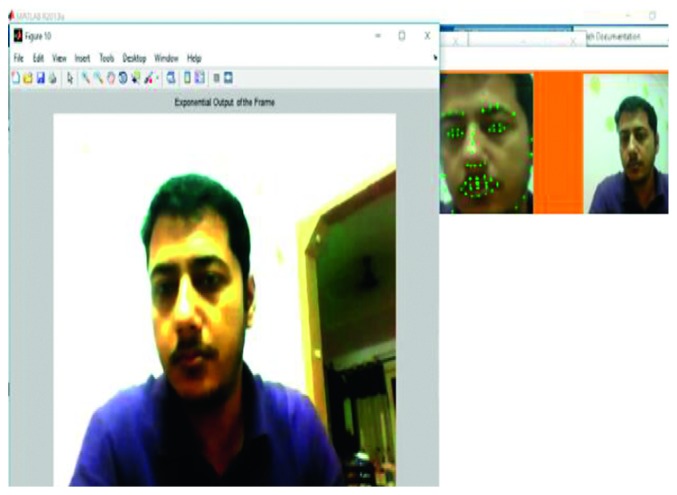
Output of exponential operation.

Figure 4 exhibits the exponential operation wherein the value of the constant *c* was set at 3 and the exponential power *γ* was set at 2. Any change in the value of the constant brought about a corresponding change in the value of the dynamic ranges of the input images. When the constant value was fixed above 3, the contrast of the bright region was enhanced. However, the best results for feature extraction were obtained at *c* = 3 and *γ* = 2.

### 3.3. Classification of Pain Face, Not Pain Face, and Neutral Face

A database was created with 22 real-time videos, containing different people of both genders and various age groups. The classification system that was employed during the course of this study employed three groups, including neutral_face, pain_face, and not_pain_face. The not_pain_face group encompassed all other basic and complex expressions. The database was prepared using peak frame rates of 30 to 50 fps for each of the facial expressions. The peak frames were the reference frames that were taken periodically. The algorithm for the current study is expressed as follows.

*Step 1*. Input the Stored Video to the System. The video could be in any format like .avi or .mp4 or .wmv or .mov. By using Application Programming Interface (API), it created an object which has all the video information.

*Step 2*. Initialisation:

- Set the number of frames to process for analysis up to 250–300 frames.
- Set the frame rate (5–15 fps).
- Set the resolution as per our requirement to either QVGA (Quarter Video Graphics Array) (320 ∗ 240) or VGA (Video Graphics Array) (640 ∗ 480).

*Step 3*. Apply Colour Conversion that converted RGB to YCbCr; i.e., frame extraction was carried out.

*Step 4*. Face detection and cropping was completed using the Viola–Jones algorithm. Cropping output was a 64 ∗ 64 matrix.

*Step 5*

- Ascertain facial landmarks in a facial video frame, and track those landmarks in a frame sequence.
- For tracking, each frame of a video was referred to as an input video frame, and the tracked facial landmarks in the previous frame were referred to as initial landmarks for the current frame.
- A hybrid model was created to detect and track the initial landmarks.
- The outputs were well-aligned landmark coordinates, head pose information, and the visibility of each landmark (66 points were considered in the present study).

*Step 6*. Extraction of Feature Points or Facial Landmark Points. In the proposed approach, six different features were extracted and they were (i) scale, (ii) pitch, (iii) yaw, (iv) roll, (v) shift of *x* coordinates from reference shape, and (vi) shift of *y* coordinates from the reference shape along with local binary patterns.

*Step 7*. Compute peak frames based on the extracted features (peak frames were the reference frames taken periodically).

*Step 8*. Prepare the database for the peak frame rates 30–50 for each facial expression.

*Step 9*. Train the model using histogram technique with reference to database.

*Step 10*. For testing every frame, apply colour conversion, face detection, and cropping and localise facial landmarks in a facial frame. Apply histogram technique and compute the Euclidean distance (ED) between the reference frame and the next frame. The decision making was based on the following rule:

  If diff < Δ1 (neutral face)
  Else diff > Δ1 but <Δ2 (not a pain face)
  Else diff > Δ2 (pain face)

where diff is the ED difference and Δ1 and Δ2 are the threshold values for classifications.

*Step 11*. Plot Receiver Operating Characteristic (ROC) curve in order to visually recognise the behaviour of the algorithm and compute the efficiency with reference to the Group of Pictures (GOP)-15 fps.

### 3.4. Training the System

Training the pain detection system involved the hybrid model and the use of the histogram technique. AAM was used to create a training mesh, followed by the application of Procrustes analysis. Procrustes analysis helped normalise the mesh and contributed to the alignment of the required shapes for the finalised model. PCA was then applied to obtain the mean shape vector, the eigenvalues, and the eigenvectors. A training data set was created with 30 to 50 peak frame rates for each of the selected facial expressions. Each face was divided into 66 facial feature points, and the resultant model was able to roughly estimate the shape of the face to find basic points. Further classification information was obtained using localised information in each facial part, which enabled the establishment of comparative locations between these parts.

The facial expression features were trained using the histogram technique, which separated the white, black, and grey pixels from the image based on the pixel intensities for each expression. Similar frequency values accumulated together, creating the histogram. Using histograms allowed for rapid and accurate analysis and helped in the stabilisation of the proposed pain detection model. This technique worked well with all images, and it was considered suitable for unsupervised training. McCann et al. had previously stated that such unsupervised techniques were preferable when there was a shortage of background information [37].

The local binary features were extracted, the magic number for the patterns was set at 9, and the Euclidean distance (ED) values for each expression were defined. The ED was considered for this study because owing to its simplicity, the required computational time was reduced. In addition to this, ED was used as the classifier for the histogram technique because it allowed for satisfactory accuracy. Histograms were plotted based on six defined features for each video in the database. Using the database that contained 22 videos as a reference, the model was trained.

**Figure 5:**
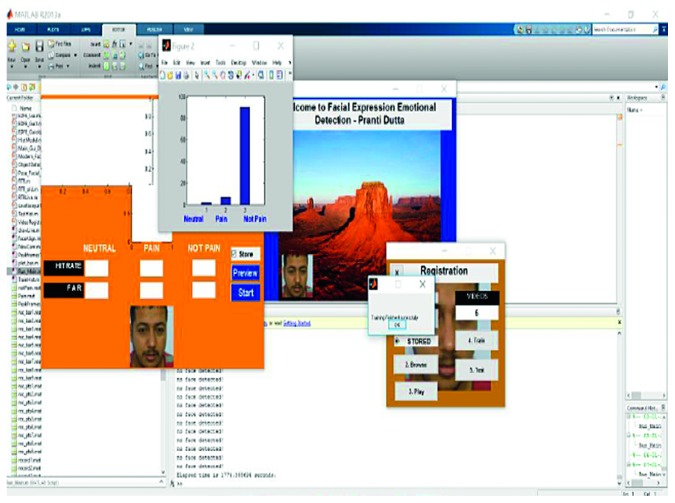
Training phase in a sample video.

Three different conditions were applied for neutral_face, pain_face, and not_pain_face with the help of soft thresholding, such that the frames were trained on a continuous basis. The training phase of a sample video is shown in Figure 5. In the figure, the pop-up window showcases how the histogram technique was used successfully to complete the training phase of a video. The data (extracted features) of the video were subjected to the histogram technique and then trained into three classifications, neutral_face (1), pain_face (2), and not_pain_face (3). These three classifications can be viewed in the central window of Figure 5 and were obtained according to the respective frames.

**Figure 6:**
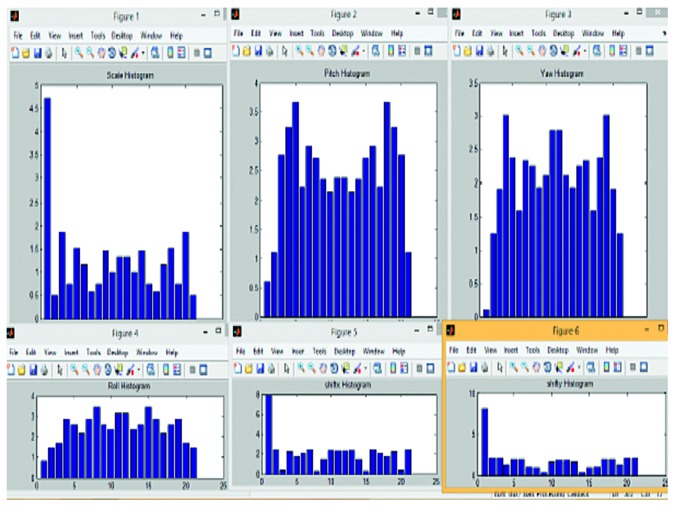
Histogram of six features of a sample video.

Following this, the histograms were plotted based on the features of the videos. The histogram of a sample video is depicted in Figure 6. In the image, the six figures display the histograms of scale, pitch, yaw, roll, shift of *x* coordinate, and shift of *y* coordinate.

### 3.5. Testing the System

The core pain detection task was evaluated during this phase. The proposed system allowed users to select any video containing a pained expression and insert it as input to the system. Faces were extracted from the video frames. Similar to the training phase, the Viola–Jones algorithm was applied to the input to allow for face detection, and the frame was cropped to a size of 64∗64 pixels. Each frame was tested using colour conversion, face detection, cropping, and the localisation of the facial landmarks in the facial frame. Continuous tracking of the facial landmarks in the facial frame was also carried out. The ED was computed between the reference video frame and the following frame. Based on the training model, three different threshold values for neutral_face, pain_face, and not_pain_face were set, and the histogram technique was applied for classification. Each frame was thus tested and classified into one of the three expressions.

**Figure 7:**
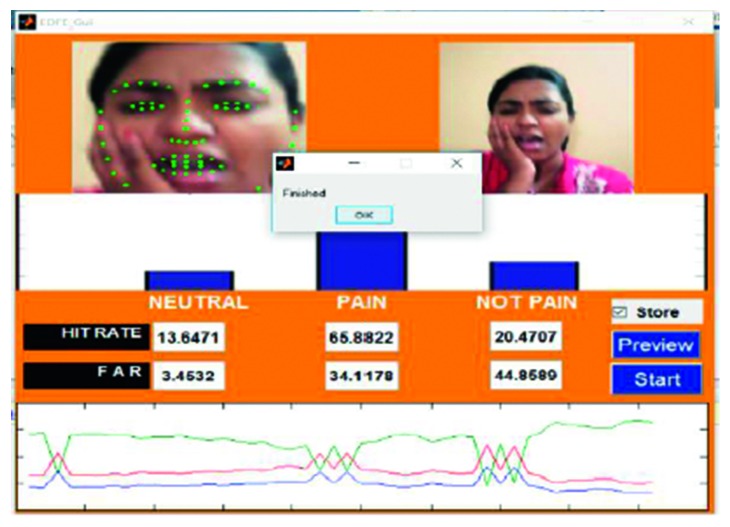
Testing phase of Video 15 in live stream.

**Figure 8:**
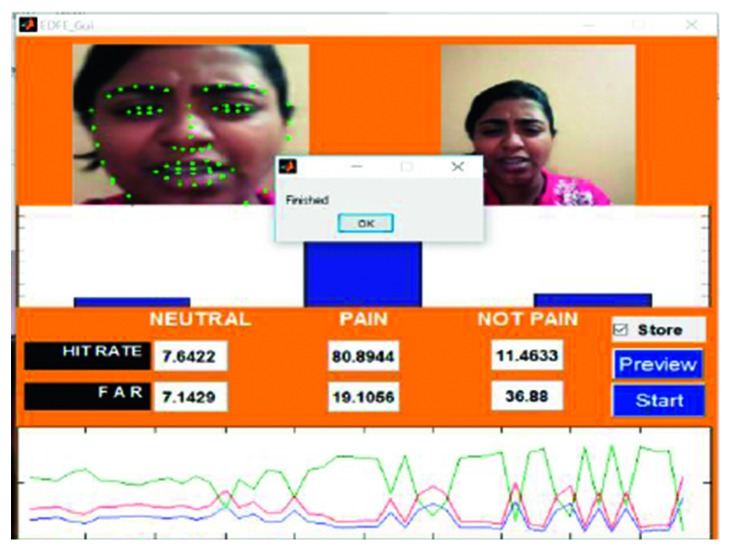
Testing phase of a stored video (Video 1).

An example of the technique is provided in Figure 7, which highlights a new sample video that was introduced into the system as live stream without any training. In this case, it was seen that there was a 65.88% hit rate for the pain_face. Another example is shown in Figure 8, which highlights a stored video that was introduced into the system without any training.

The described algorithm was executed in MATLAB 2017a, using C on an Intel CORE i5 Processor Windows 10 workstation, which had a processing power of 4 GB RAM. The videos that were captured in this study were recorded at varying distances from the camera, at different angles, using variable lighting conditions and resolutions, and with different contexts and mixed expressions recorded along with pain. The database used for analysis consisted of videos containing a varying set of subjects. This included four adult women, nine adult men, and one child. Cameras included in this study to capture real-time videos included laptop web cameras, mobile phone cameras, a tablet camera, and digital cameras.

### 3.6. Training and Testing with Multilayer Back propagation Neural Network (MLBPNN)

**Figure 9:**
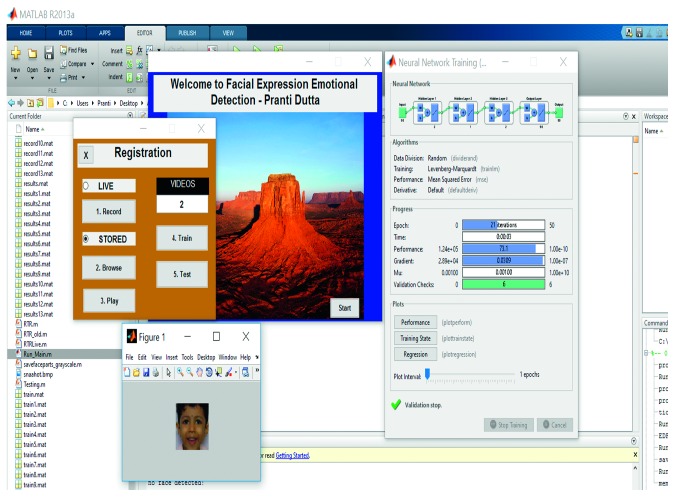
Training part of a sample video using multilayer backpropagation neural network.

**Figure 10 fig10:**
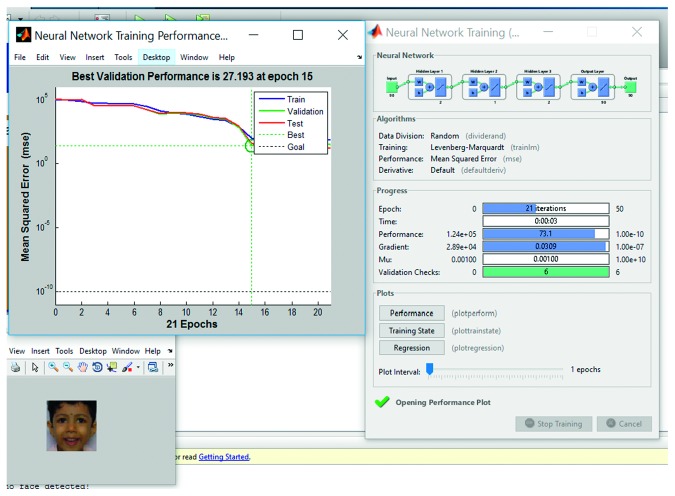
Training performance of MLBPNN. It shows 27.193 is the best validation performance at epoch 15.

**Figure 11 fig11:**
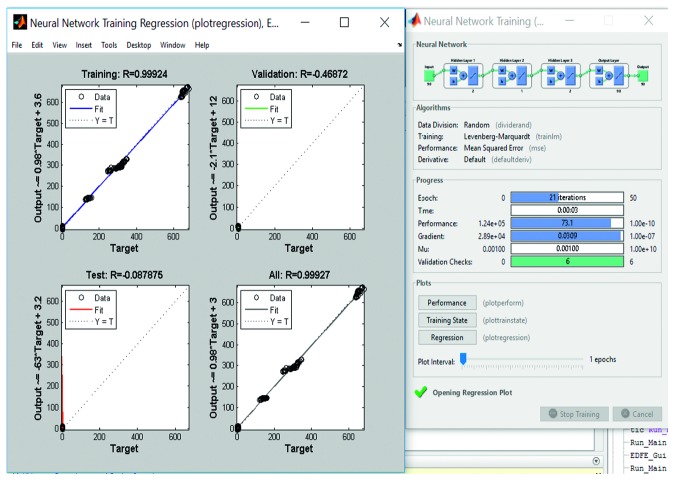
Training part of a sample video using multilayer backpropagation neural network (showing training performance).

**Figure 12:**
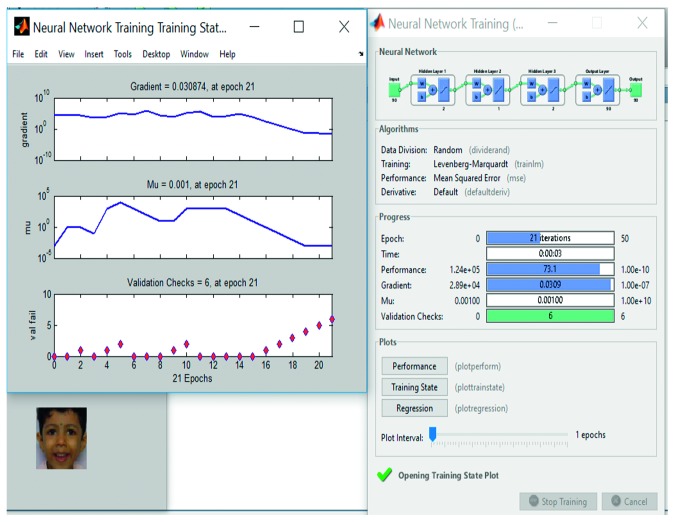
Training part of a sample video using multilayer backpropagation neural network (showing training state).

Despite protracted training time, the neural network based model is always a good fit for adaptive learning and is a preferred technique for expression/emotion recognition. Apart from histogram technique for training the system, MLBPNN was applied as an approach to detect pain in the present study to compare the processing speed and accuracy of the proposed algorithm. The proposed MLBPNN composed of one input layer, three hidden layers with five nodes, and one output layer. The five nodes were arranged in such a way that two nodes are in the first hidden layer and one node and two nodes in the second and third hidden layer, respectively. During the training period, frame-by-frame results were taken such that for every 90 input frames, 90 output values divided into three results (neutral_face, pain_face, and not_pain_face). Use of three hidden layers considerably increased the training time without bringing in significant increase in accuracy compared to the histogram technique. The training period of a sample video along with best validation performance, training performance, and training state are shown in Figures 9 [Fig fig10] [Fig fig11]–12, respectively.

## 4. Results

Information from the 22 videos was collected and analysed to understand the effectiveness of the developed model. A Receiver Operating Characteristic (ROC) curve was used to exhibit the accuracy of the frame-level detection of pain in individual videos. Range was used to represent the frame-by-frame accuracy percentage. This parameter was the plot of the relation between the False Acceptance Rate (FAR), which represented false positives and Hit Rates (HR), which represented true positives. HR represented the accurate detection of pain in the video sequences, whereas FAR signified the proportion of videos with not_pain that were incorrectly identified as pain during the course of the study. The output from each of the videos was analysed. In each case, it was seen that the *x*-axis represented the frame index in the video and the *y*-axis represented the predicted pain score. Dotted arrows indicated the correspondence between the image frames (top row) and their predicted classification (neutral_face, pain_face, and not_pain_face) scores. All images that contained expressions that could neither be classified as pain or neutral were classified into the not_pain category. Individual colours were used for each classification for easier identification and visualisation, with blue used for neutral_face, green used for pain_face, and red used for not_pain_face (some of the images included in this study are only for representative purposes and are a combination of pained as well as painless expressions).

A frame from the first video is shown in Figure 8. Video 1 was captured using a video camera, with the footage stored in an .mp4 format. The entire video was 22 seconds in length and 3.02 MB in size. During the course of the video, the subject maintained a smiling face for the first 9 seconds and a pained expression for the remaining 13 seconds. The accuracy for the detection of the pain_face was 80.89%–86.41% and for the smile, which was categorised as a not_pain_face, the accuracy was 80.45%–86.85%.

**Figure 13:**
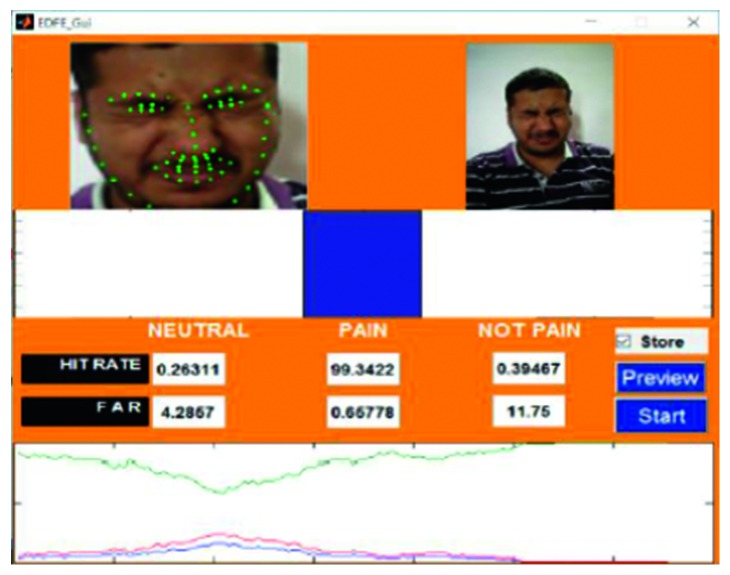
Video 4 recorded using a digital camera.

Frame from video 4 is shown in Figure 13, along with the analysis. The video was captured using a digital camera, and the footage was stored in a .wmv format. The length of the video was 49 seconds, and the size was 102.2 MB. The subject of the video maintained a neutral expression for the first 10 seconds, a surprised face for 15 seconds, and a pained face for 24 seconds. The accuracy for the detection of the pain_face was found to be 91.12%–99.34%, it was 87.88%–92.22% for neutral_face, and for the not_pain_face, which was the classification provided to the surprised face, the accuracy was 74.99%–78.54%.

**Figure 14:**
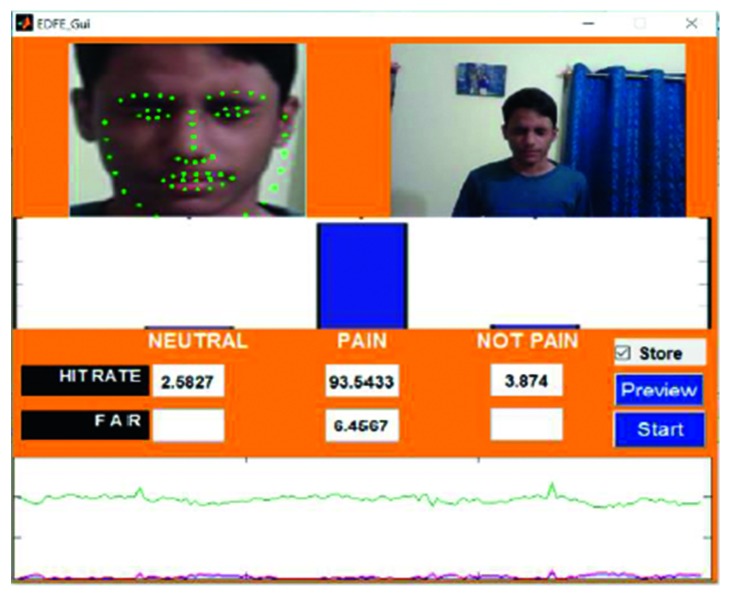
Video 5 recorded with a mobile phone camera.

Video 5 was recorded using a mobile camera, and a substantial distance was maintained between the subject and the camera. Additionally, the recording was carried out under poor lighting conditions with noisy background. The footage, which was recorded in an .mp4 format, was 28 seconds long and 7.95 MB in size. The subject maintained a neutral expression for the first 10 seconds and a pained expression for the next 18 seconds. Overall, the accuracy of detection for the neutral_face was 89.97%–97.02%, and that of the pain_face was 90.00%–98.76%. The output from video 5 can be seen in Figure 14.

**Figure 15:**
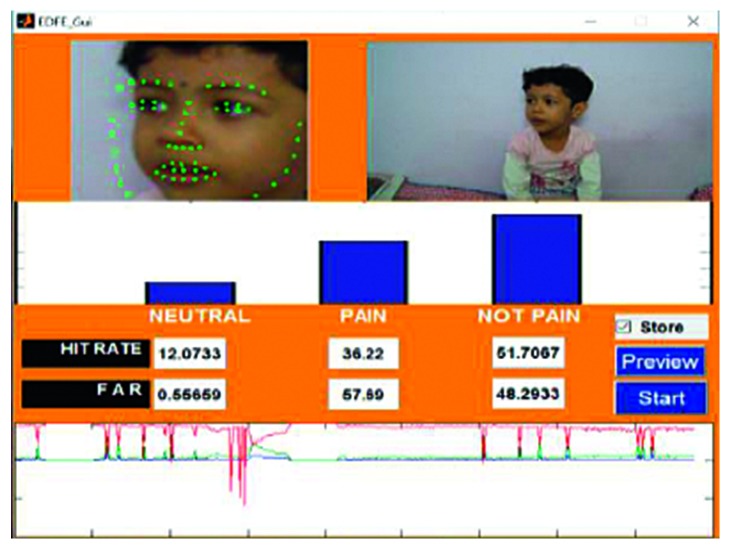
Video 6 recorded using a digital camera.

Video 6 was recorded using a digital camera, specifically incorporating a change of angle in the head position of the subject. The footage of the video was recorded in an .mov format and was 30 seconds in length and 100.2 MB in size. During the video, the subject maintained a smiling expression for the first 8 seconds, a neutral face for the next 15 seconds, and a pained expression for the last 7 seconds. The accuracy for the neutral_face was 51.70%–55.34%, for the pain_face was 69.11%–74.29%, and for the smiling expression, which was classified as a not_pain_face, the accuracy was 84.45%–86.85%. A frame and the output from video 6 are shown in Figure 15.

**Figure 16:**
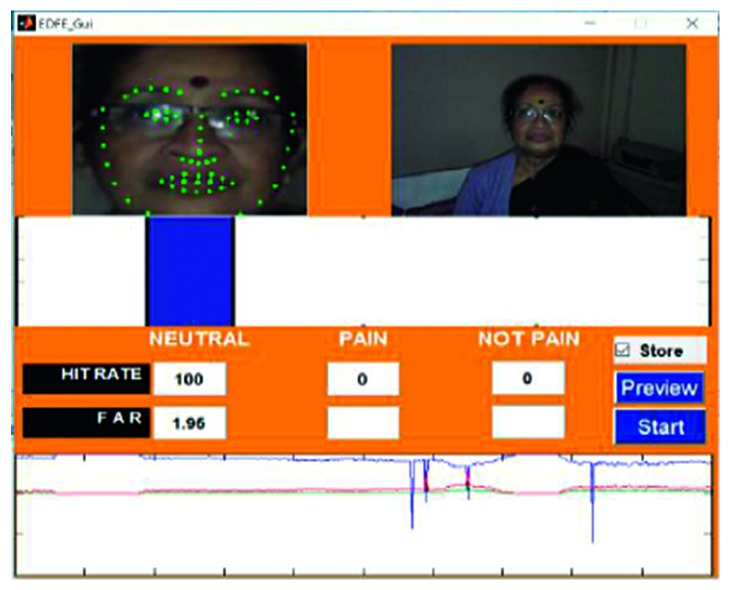
Video 10 recorded using a laptop web camera.

The analysis of video 10 is depicted in Figure 16. This video was recorded using a laptop web camera under poor lighting conditions. The footage was stored in an .avi format. The recorded video was 20 seconds long and 700 MB in size. The subject of the video maintained a neutral expression for the first 10 seconds and a pained expression for the remaining 10 seconds. Overall, the accuracy of the detection system was found to be 99.97%–100% for the neutral_face and 85.55%–91.72% for the pain_face.

**Figure 17:**
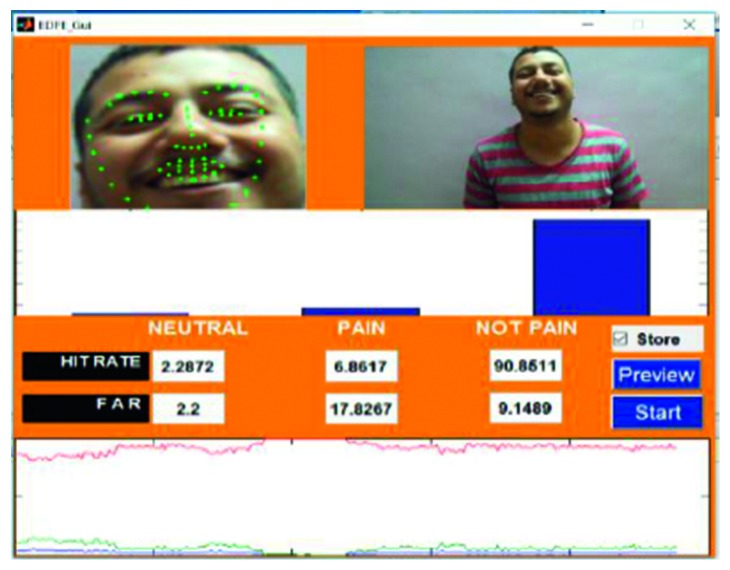
Video 21 recorded using a laptop web camera.

Video 21, which is represented in Figure 17, was recorded using a laptop web camera in an .avi format, and the subject changed their position (head is upward). The video was 50 seconds in length and 352.2 MB in size. The subject maintained a neutral expression for the first 5 seconds, a smiling expression (not_pain_face) for the next 10 seconds, an angry expression for 5 seconds, a pained expression for 25 seconds, and another neutral face for the last 5 seconds. Overall, the accuracy for the detection of the neutral_face was found to be 77.67%–81.34% and 79.11%–84.29% for the pain_face and for the not_pain_face (smiling and angry), and the accuracy was 89.45%–90.85%.

**Figure 18:**
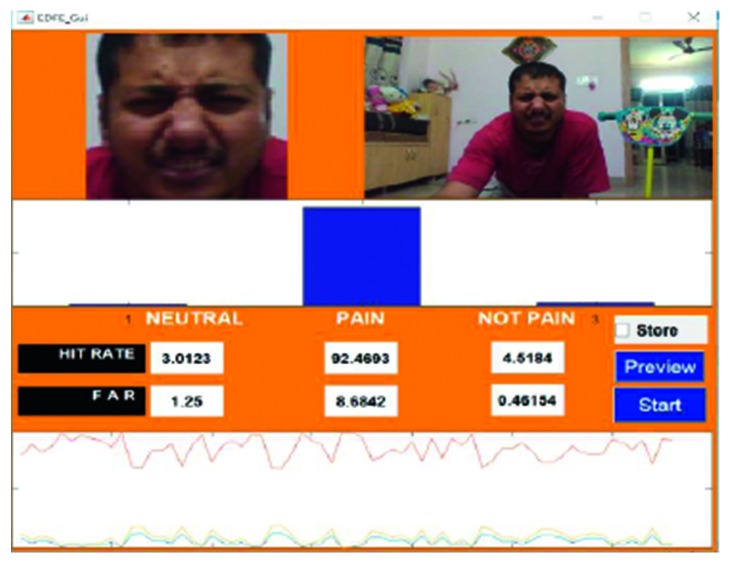
Video 2 recorded in a live environment using a laptop web camera.

**Figure 19 fig19:**
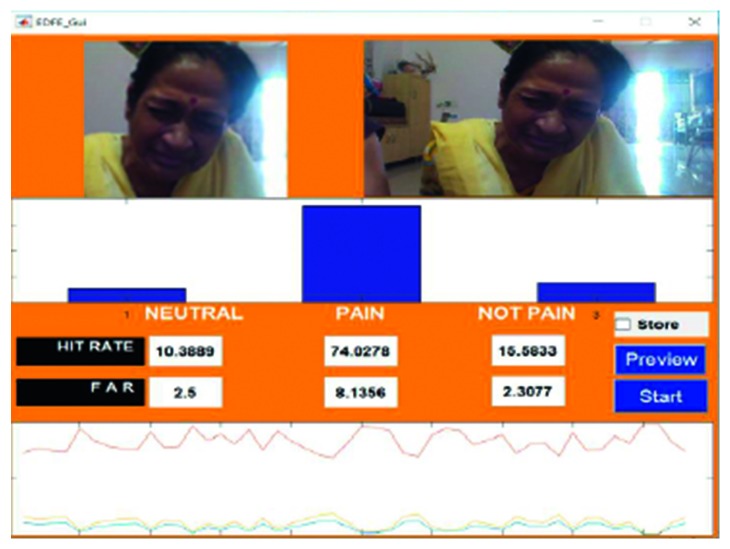
Video 3 recorded in a live environment using a laptop web camera.

**Figure 20:**
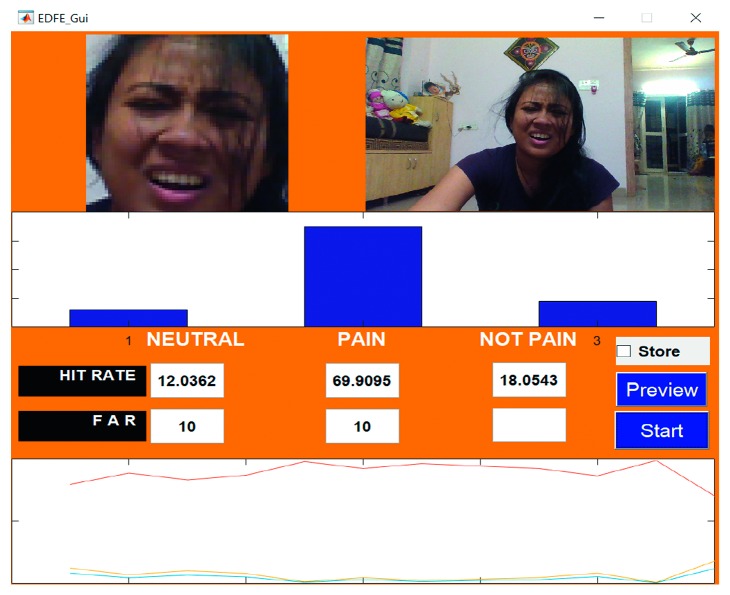
Video 22 recorded in a live environment using a laptop web camera.

Figures 18 [Fig fig19]–20 represent the output from three of the live-streamed videos. The videos were recorded using a laptop web camera. In the live-streamed videos, the external lighting conditions were observed to fluctuate (owing to changes in the sunlight as well as in indoor lighting). The videos were 2 minutes, 5 minutes, and 6 minutes 17 seconds in length and 72.04 MB, 116.5 MB, and 194.5 MB in size. Overall, the accuracy for the detection of the pained expression was found to be 90.47%–92.46%, 74.02%–82.23%, and 69.90–74.87%.

### 4.1. Comparison with a Standard Database

To increase the reproducibility of this technique, the proposed hybrid model was tested on the homemade database that was described in this experiment and on a standard UNBC-McMaster Shoulder Pain Database. The standard database contains 200 sequences spanning across 25 subjects, which contributes to a total of 48,398 images. The given database was selected owing to the large volume of representative data that was made easily available to researchers. The database also had a lot of head pose variations across both genders, thus making it an ideal database to test the proposed algorithm.

**Figure 21:**
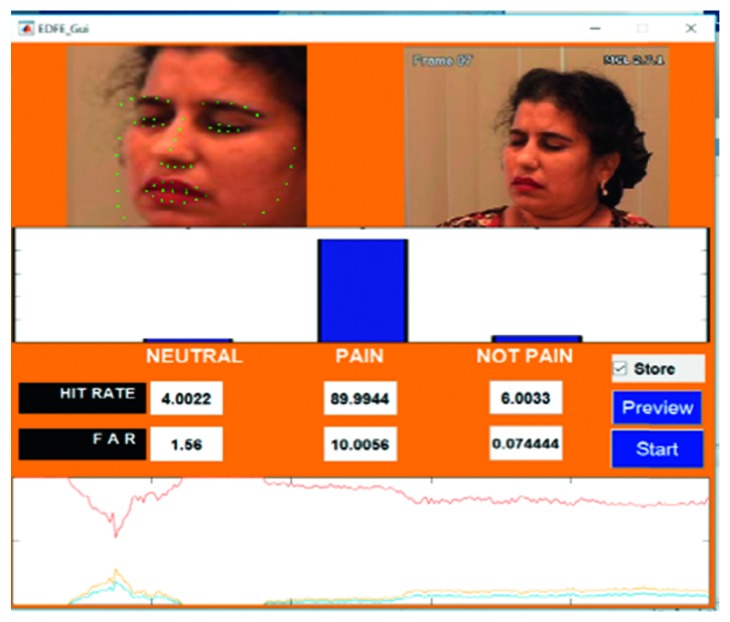
Video hs107t1afaff.

**Figure 22:**
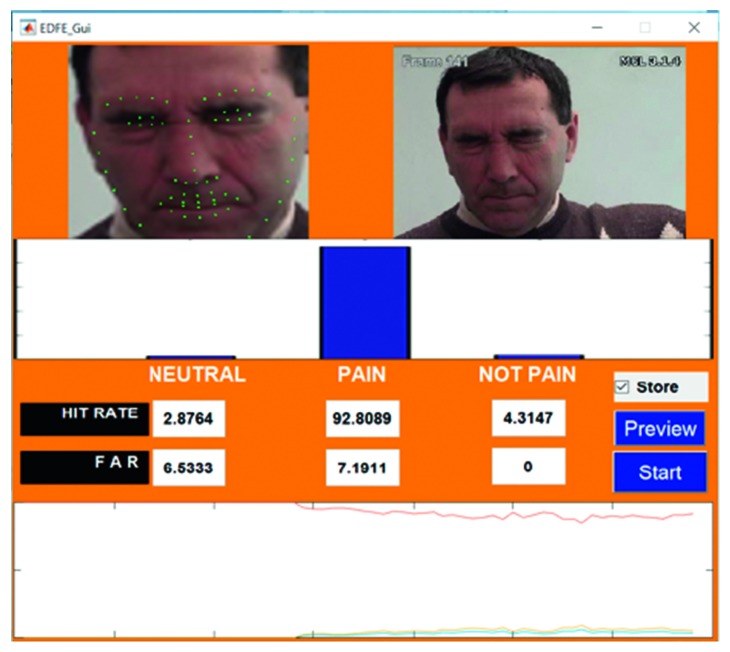
Video ak064t1aaaff.

For this study, only relevant image sequences containing pained expressions and other expressions were selected. These image sequences were then converted into videos. Thus, a total of 24 videos in an .avi format were extracted of 22 subjects. Overall, when the proposed hybrid model was used on the UNBC-McMaster Shoulder Pain database, it was found that the accuracy was more than 95%. Images of the pained expressions from the UNBC database that were obtained by using the hybrid are shown in Figures 21 and 22, with further samples shown in Appendix.

## 5. Discussion

The training time, test time, and HR of all 22 of the recorded videos are displayed in Table 1, along with information related to the number of frames, the format of the video, and the size. Out of the 22 videos, videos 2, 3, 8, 15, and 22 were all recorded from a live stream.

From the collected data, it was evident that the proposed model had a minimum accuracy of 65.88%. The compiled HR values ranged from 65.88% to 100%. Based on these findings, it was observed that using suitable conditions, automated pain recognition systems to detect and identify pain had the potential to be extremely efficient. This highlighted their usefulness and corresponded to earlier studies [38–40].

When the findings were analysed, it was found that there was no clearly established trend between HR and the format, size, number of frames, and the training or testing times. The researcher interpreted the variations in the HR to be a result of the positioning of the head and the contextual information that was present along with the subject. Slight variations in terms of the distance between the subject and the recording device, as well as the nature of the recording device could have contributed to the variation in HR. Lucey et al. and Werner et al. had discussed the differences that arose in the accuracy of pain detection owing to the variations in facial geometry and expression of pain [7, 8]. Tian et al. had also elaborated on the different intensities of facial expressions [41]. Thus, it could be assumed that those individuals that exhibited low-intensity facial expressions showed a lower HR. The range of HR that was encountered in the given study could be explained as a result of these findings.

The lowest HR value was found in video 15 (Figure 7), which was a live-streamed video. The accuracy in this case was reported to be 65.88%–69.11%. The HR for the other live-streamed videos was higher, with all the videos recording more than 70% accuracy. In fact, video 2 showed a HR value of around 90%. The quality of the live-streamed video had an impact on the overall HR value. Videos that were recorded in a live environment had to contend with fluctuating lighting conditions, heightened noise levels, and continuous changes in the positioning of the head, all of which had the potential to impede the frame extraction. The live-streamed videos also had other obstacles in the form of increased background disturbances and were recorded using a laptop web camera. Video 15 had an additional hindrance in terms of an occlusion, which was introduced owing to the placement of the subject's hand. This may have prevented frame extraction and impacted the HR. Regardless of these obstacles, the HR of the live-streamed videos remained above 65% in all cases, which indicates the potential of the current model.

Lighting and scaling are also important factors that influence the HR. The findings in the present study indicated that occlusions decreased the HR, which was similar to previous studies [42]. An example of this was seen in video 15, and the researcher assumed that the appearance points on the occlusion got included with the feature points on the face. The extraction that occurred was faulty, and the HR was reduced. Lower accuracy was noted for faces that were not looking directly at the recording device, which matched the findings stated by Rupenga and Vadapalli [40]. Sikka et al. reported that motion in front of the camera while recording reduced the accuracy of the system [43]. Thus, a combination of all these factors, including lighting, head position, noise, occlusions, and scaling, need to be taken into account when constructing the final, robust model, as they were found to affect the efficiency of the algorithm and model. However, the researcher found that the HR was high even in the cases of poor lighting, as was seen in videos 5 and 10, indicating success for the proposed model.

The proposed model and technique were useful in reducing the size and the memory required for storage.  Table 2 provides a summary of the storage information, which was described using the four operations and a comparison of their reduction of storage. The original memory size occupied by the video has been compared to the reduced space needed for the stored matrix images (video frames). From Table 2, it is clear that the proposed hybrid model allowed for a substantial decrease in the space needed for the storage of images, which was an important benefit of the system. In this table, the memory required for the storage of each video during feature extraction was compared for the hybrid model and the point operations. The hybrid model that was utilised for this study incorporated a patch-based approach for handling the images and data. As a result of this method, the overall system needed more memory, which could lead to memory overloading, especially in cases of larger video inputs and live buffering. Image algebra was introduced to overcome these limitations. Point operations that were introduced at this junction depended on the input intensity at that point and did not involve explicit spatial memory. Using image algebra, in this manner improved the visual appearance of the image and increased the ease of feature extraction. The point operations that were utilised along with the hybrid model required less memory for storage than the hybrid, which served as a unique feature of this study.

The logarithmic operation that was employed enabled an improved output by allowing for better feature extraction. The operation reduced the values of bright pixels and concurrently enhanced the values for dark pixels, allowing for better contrasts. This point operation also allowed for a restricted recording and representation of an array of high-level grey scale intensity information as equally high-level output.

The most efficient reduction was seen owing to the use of the logarithmic operation, but similar reductions were observed for the remaining operations. The reduction in storage increased the processing speed and reduced the software and hardware requirements for the procedure. Image algebra, when applied separately to facilitate feature extraction, allowed for improved reduction of memory (up to 40%–55%). Thus, the application of the proposed model, along with image algebra and the point operations, allowed for numerous benefits.

## 6. Conclusion

The present study proposed the use of a hybrid model and image algebra to identify the expression of pain on subjects' faces, using videos and real-time streamed data as input. The process met with success as it was able to accurately distinguish between pain and nonpain expressions, despite being subjected to a number of unfavourable conditions such as poor lighting, changes in the head position, introduction of occlusions, and the incorporation of pain among a sequence of other expressions. Accurate identification was noted regardless of the mixture of a pained expression with other expressions, and a high rate of success was observed even in the case of poor lighting, which was a marked advantage of this system. The presence of occlusions and changes in the positioning of the head resulted in the reduction of the accurate prediction of pain, but this was an expected finding. Despite the current study not relying on any in-built databases and only using raw data, the accuracy rates remained above 65% for all analyses, highlighting the efficacy of this model. For real-time computation, speed is a concern. ED with the histogram technique does not require much computation time but trade off may lose accuracy. In this work, we are getting more than 90% accuracy using the histogram technique. Whereas MLBPNN gave almost same accuracy as well, but it takes more time to execute as compared to ED on the histogram technique.

Future studies could involve the incorporation of additional file formats, as the current study was limited by the formats that could be supported by MATLAB. Additionally, each analysis consisted of only a single subject. Future analyses could include the study of multiple subjects and the comparison with more preexisting databases. While the current study included only two point operations, further experiments can be conducted with additional point operations to study the efficacy. Greater numbers of subjects could be studied using the model, with increased variety in the different positions of the head and increased training time for the system. The current study relied solely on the use of the histogram technique and MLBPNN, but future research should include the implementation of other approaches as classifiers in image processing, including the use of different neural networks methods and mathematical techniques, such as partial differential equations. Histogram techniques use the concept of uniform pixel distribution [44]. A value for the mean pixel distribution is calculated, and the distance between the mean value and the individual pixel is utilised to enhance the contrast of the image. Neural networks, on the other hand, are designed based on biological nervous systems. Individual units similar to neurons are used, with the output from one unit serving as the input to another unit. Mathematical models can also be used for analysis, especially prevalent types of image algebra and linear algebra.

Findings from the present study offer a useful foundation for the development of a cost-effective system that can be used to detect pain in hospitals, clinical environments, and home-care settings, thus presenting several implications for medical professionals and health-care practitioners.

## Figures and Tables

**Table 1:** Performance analysis of 22 videos.

Video number	Video size (MB)	Video format	Number of frames	Training time	Testing time	HR (pain) in range (%)
1	3.02	.mp4	140	7 m 5 s	2 m	80.89–86.41
2 (live)	72	.avi	232		2 m	90.47–92.46
3 (live)	116.5	.avi	512		5 m	74.02–82.23
4	102.2	.wmv	1011	15 m	4 m	91.12–99.34
5	7.95	.mp4	200	4 m 56 s	1 m	90.00–98.76
6	100.2	.mov	700	9 m	3 m 36 s	69.11–74.29
7	167.4	.avi	1072	16 m	7 m	82.92–87.85
8 (live)	234	.avi	1598		8 m 53 s	70.17–78.76
9	9.47	.mp4	223	5 m 18 s	1 m 53 s	87.77–90.00
10	700	.avi	2236	20 m 19 s	5 m	85.55–91.72
11	7.9	.mov	70	20 s	5 s	91.00–93.33
12	28.8	.mov	220	45 s	11 s	78.76–82.92
13	50	.avi	250	9 m 18 s	2 m 40 s	94.33–100.00
14	30.2	.mov	231	3 m 39 s	1 m 11 s	92.09–97.56
15 (live)	136.1	.avi	754		5 m 30 s	65.88–69.11
16	42.6	.wmv	470	7 m	2 m 43 s	79.14–81.21
17	46.1	.wmv	501	8 m 13 s	3 m	83.44–87.11
18	50.4	.wmv	540	9 m 27 s	3 m 11 s	75.34–86.21
19	10.1	.mp4	219	11 m 7 s	1 m	67.45–75.46
20	30.2	.avi	209	8 m 43 s	1 m 34 s	67.11–70.30
21	352.2	.avi	913	15 m 19 s	5 m	79.11–84.29
22 (live)	194.5	.avi	1041		6 m 17 s	69.90–74.87

**Table 2:** Storage summary in matrix.

Serial no.	Name of the operation in feature extraction	Elapsed time (in seconds)	Storage summary (in original) based on all videos	Storage summary (in matrix)	Reduction of storage (in %)
1	Hybrid model	1.921336–2.063168	2.08 MB–700 MB	0.987 MB–212 MB	52.54–69.71
2	Logarithm	0.752480–1.630598		0.45 MB–89.12 MB	78.37–87.29
3	Exponential	0.749532–1.296239		0.46 MB–117.1 MB	77.88–83.28

**Table 3:** Performance analysis of 24 videos (UNBC database).

Sl. no.	Video name (.avi)	Video size (MB)	Number of frames	Training time	Testing time	HR (pain) in range (%)
1	aa048t2aeaff	2.09	173	9 m 22 s	39 s	90.23–95.88
2	ak064t1aaaff	3.94	396	21 m 11 s	1 m 52 s	92.80–96.12
3	bg096t2aaaff	2.76	232	12 m 36 s	53 s	90–93.33
4	bm049t2afaff	3.65	351	18 m 50 s	1 m 35 s	94.33–98.65
5	bn080t1afaff	3.97	381	20 m 30 s	1 m 46 s	97.11–99.9
6	ch092t2aiaff	5.39	374	19 m 43 s	1 m 26 s	89.42–95.33
7	dn124t1aiaff	4.11	420	22 m 40 s	1 m 36 s	90.22–93.33
8	dr052t2aiaff	4.76	467	24 m 47 s	1 m 47 s	88.99–92.22
9	fn059t2aiaff	4.25	367	19 m 56 s	1 m 24 s	87.45–94.22
10	gf097t1aaaff	5.66	516	27 m 22 s	1 m 59 s	85.88–90.67
11	hs107t1afaff	3.15	285	15 m 19 s	1 m 05 s	89.99–93.44
12	hs107t1afunaff	3.32	263	14 m 01 s	1 m 01 s	90.23–93.76
13	hs107t2aaaff	4.04	411	21 m 37 s	1 m 34 s	89.09–94.54
14	ib109t1aiaff	2.62	321	17 m 10 s	1 m 14 s	92.22–96.06
15	jh123t1aeaff	3.7	358	19 m 08 s	1 m 22 s	91.03–96
16	jk103t2aiaff	3.24	300	15 m 32 s	1 m 09 s	93.33–98.09
17	jl047t1aiaff	5.36	464	24 m 11 s	1 m 43 s	96.41–99.98
18	kz120t2aaaff	1.38	134	7 m 14 s	31 s	92.02–95.33
19	ll042ll042t1aiaff	1.68	168	8 m 12 s	38 s	97.76–100
20	mg066t1aaaff	4.53	405	21 m 58 s	1 m 33 s	90.77–93.90
21	mg101t1aiaff	5.8	615	32 m 17 s	2 m 21 s	94.22–98.33
22	nm106t1afaff	3.99	351	18 m 02 s	1 m 21 s	97.12–100
23	th108t2afaff	2.64	277	14 m 13 s	1 m 04 s	92.08–96.73
24	vw121t1aaaff	2.04	179	9 m 54 s	41 s	96.42–99.33

## Data Availability

The data used to support the findings of this study are available from the corresponding author upon request. As there are no readily available video databases that capture facial pain, the authors have created a video database in their personal capability to carry out the research. While the subjects in the video have given explicit permission to use their videos for their research purpose, the same cannot be published or used beyond their research or for any commercial purpose.

## Appendix

The performance analysis of the 24 videos from the UNBC database is shown in Table 3. The table contains information about the video names, sizes, number of frames, training times, testing times, and hit rates.

## Supplementary Materials

Supplementary Materials Figure X: training part of a sample video using multilayer backpropagation neural network. Figure Y: training part of a sample video using multilayer backpropagation neural network (showing training performance). Figure Z: training part of a sample video using multilayer backpropagation neural network (showing training state). Figure XX: training part of a sample video using multilayer backpropagation neural network (showing regression). Figure XY: testing part of a sample video using multilayer backpropagation neural network (pain_face). Figure YY: testing part of a sample video using multilayer backpropagation neural network (not_pain_face). Figure ZZ: testing part of a sample video using multilayer backpropagation neural network (neutral_face).
