# Supplementary material for: Facial Pain Expression Recognition in Real-Time Videos
Source: J Healthc Eng. 2018 Oct 30;2018:7961427. doi: 10.1155/2018/7961427 (PMC6231392; doi:10.1155/2018/7961427)
Supplement: Supplementary Materials — Figure X: training part of a sample video using multilayer backpropagation neural network. Figure Y: training part of a sample video using multilayer backpropagation neural network (showing training performance). Figure Z: training part of a sample video using multilayer backpropagation neural network (showing training state). Figure XX: training part of a sample video using multilayer backpropagation neural network (showing regression). Figure XY: testing part of a sample video using multilayer backpropagation neural network (pain_face). Figure YY: testing part of a sample video using multilayer backpropagation neural network (not_pain_face). Figure ZZ: testing part of a sample video using multilayer backpropagation neural network (neutral_face). [file 7961427.f1.pdf]

Multilayer Back Propagation Neural Network: For pain detection, there is another approach called Multilayer Back Propagation Neural Network is applied in the proposed work. Fig. X shows the training period of a sample video. In this method, frame by frame results are presented. i.e. for every 90 input frames there are 90 outputs with three results (neutral_face, pain_face and not_pain_face). There are three hidden layers with five nodes in the proposed MLPBNN. Two nodes in the first hidden layer, one node in the second hidden layer and two nodes in the third hidden layer.

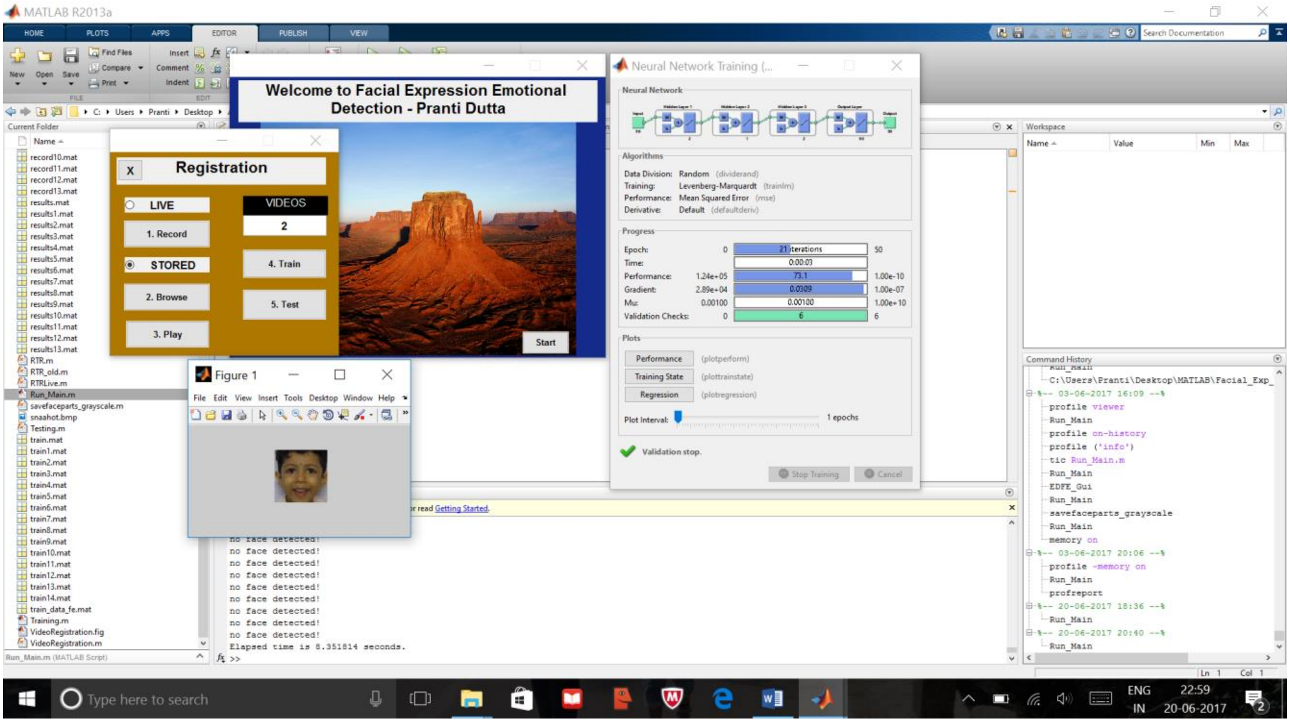

Figure X: Training part of a sample video using Multilayer Back Propagation Neural Network

Fig. Y represents the training performance of MLPBNN. It shows 27.193 is the best validation performance at epoch 15.

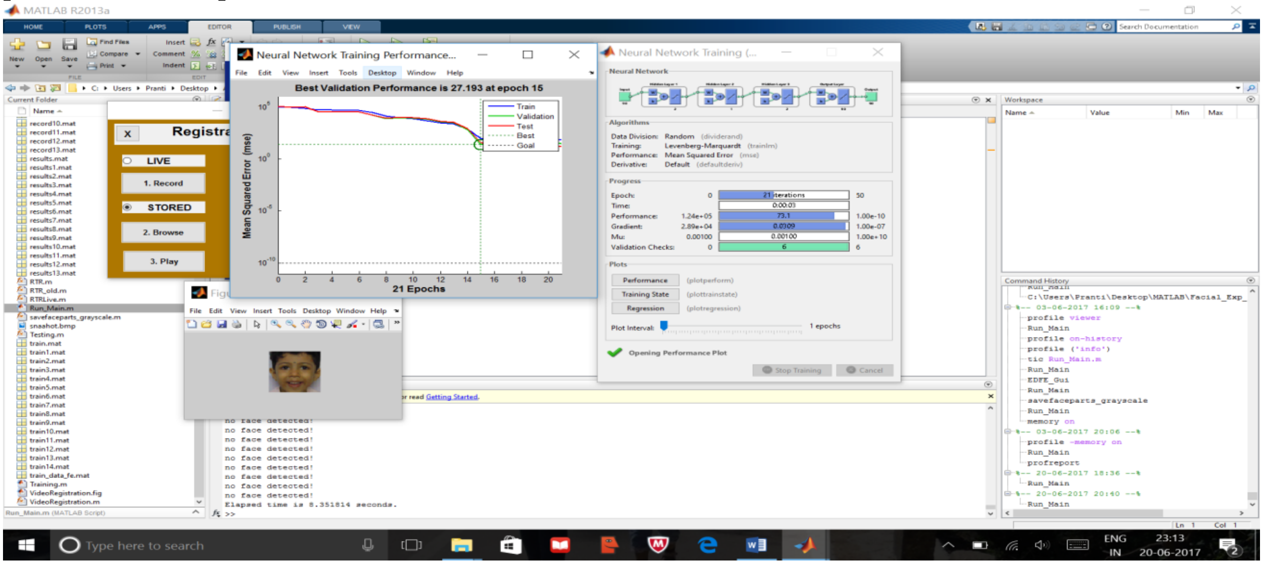

Figure Y: Training part of a sample video using Multilayer Back Propagation Neural Network (showing training performance)

Fig. Z represents training state of MLBPNN in terms of three aspects. Firstly, variation in gradient coefficient with respect to number of epochs (here epoch= 21). The value of gradient coefficient at epoch number 21 is 0.030874. From figure it can be seen that gradient value goes on decreasing (tends to zero) with increase in number of epochs. Secondly, adjusted value of mu. Depending on the particular problems the value of mu is keep changing. It is based on the diversity nature of the system i.e. converging or diverging. In this work, it is in range of 10^5 to 10^{-5}. Thirdly, validation check of the network. Here, validation check is 6 out of 6 at epoch 21.

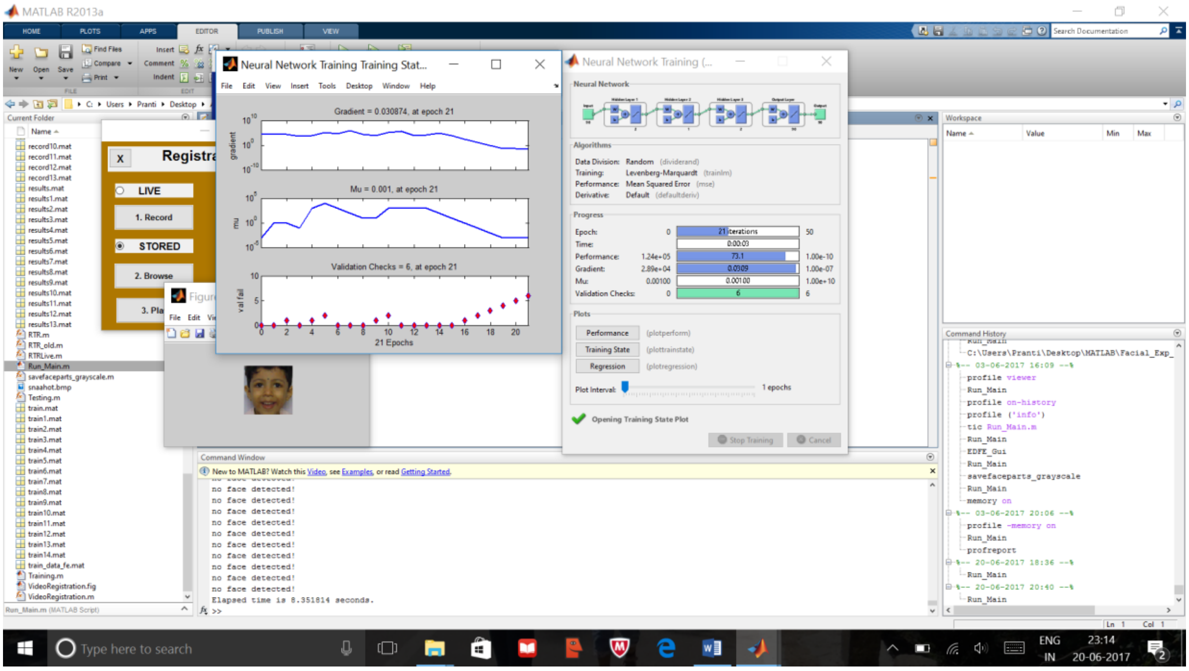

Figure Z: Training part of a sample video using Multilayer Back Propagation Neural Network (showing training state)

Fig. XX shows four plots representing the training, validation, testing and together all. For training, R = 0.99924, which indicates that there is approximately an exact linear correlation between outputs and targets. For testing, R=0.087875 and for validation, R=0.46872.

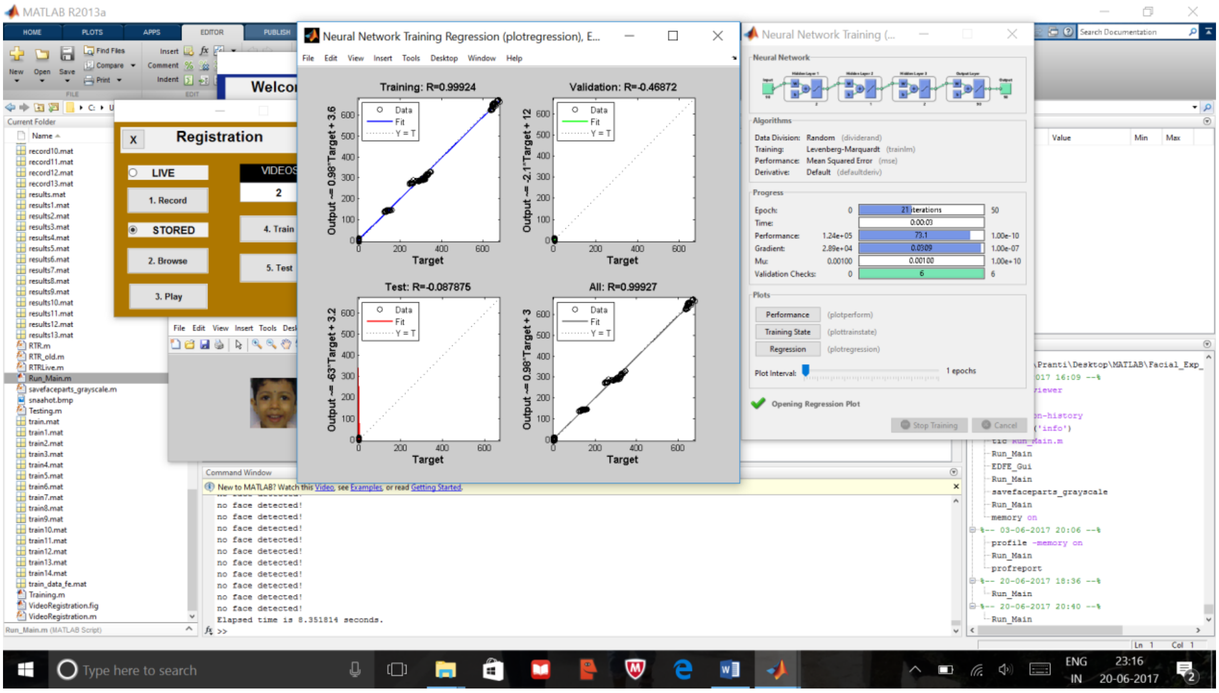

Figure XX: Training part of a sample video using Multilayer Back Propagation Neural Network (showing regression)

Fig. XY exemplifies the testing period of the given sample video. The tested pain detection hit rate is 84.83. Fig. YY and Fig. ZZ shows the tested pain_face and neutral_face with their respective hit rates 84.79 and 85.27.

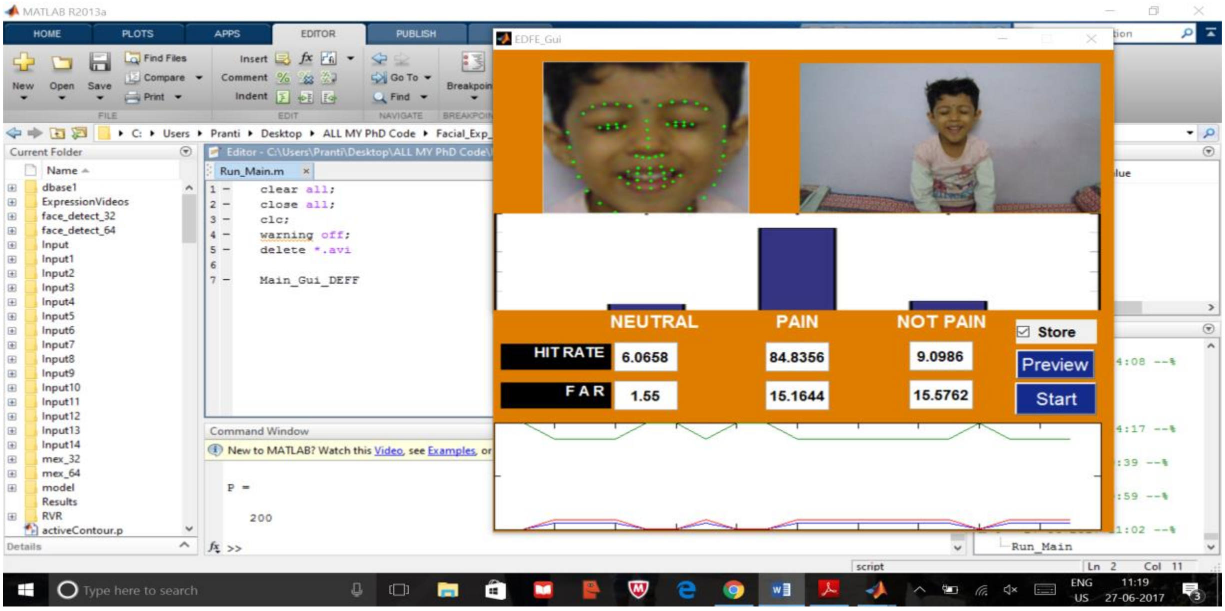

Figure XY: Testing part of a sample video using Multilayer Back Propagation Neural Network (pain_face)

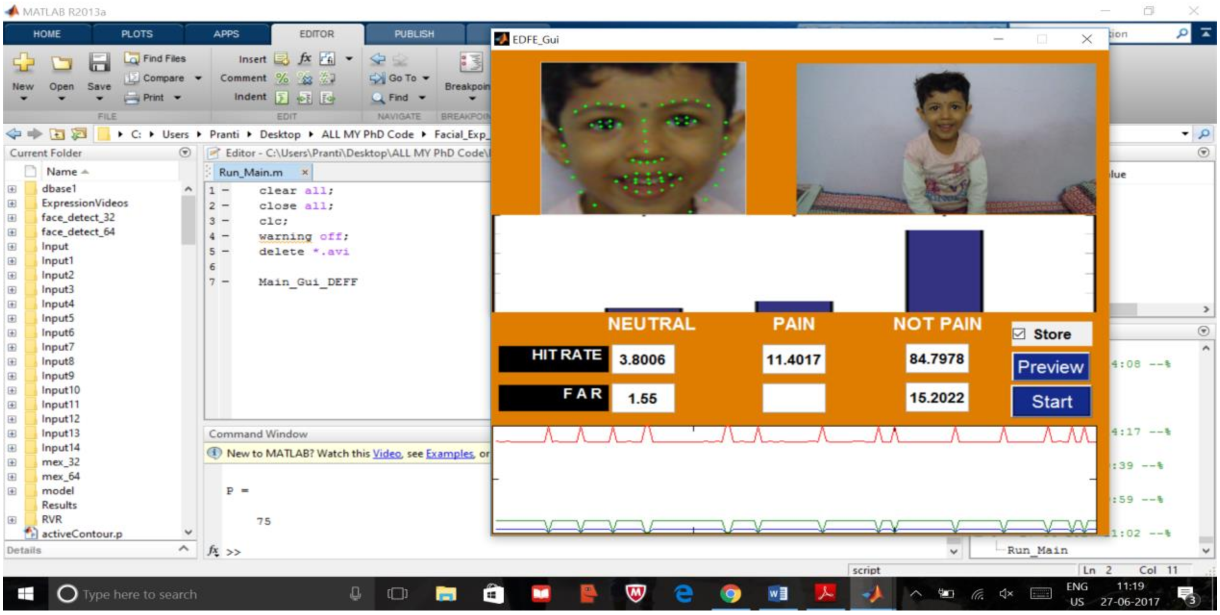

Figure YY: Testing part of a sample video using Multilayer Back Propagation Neural Network (not_pain_face)

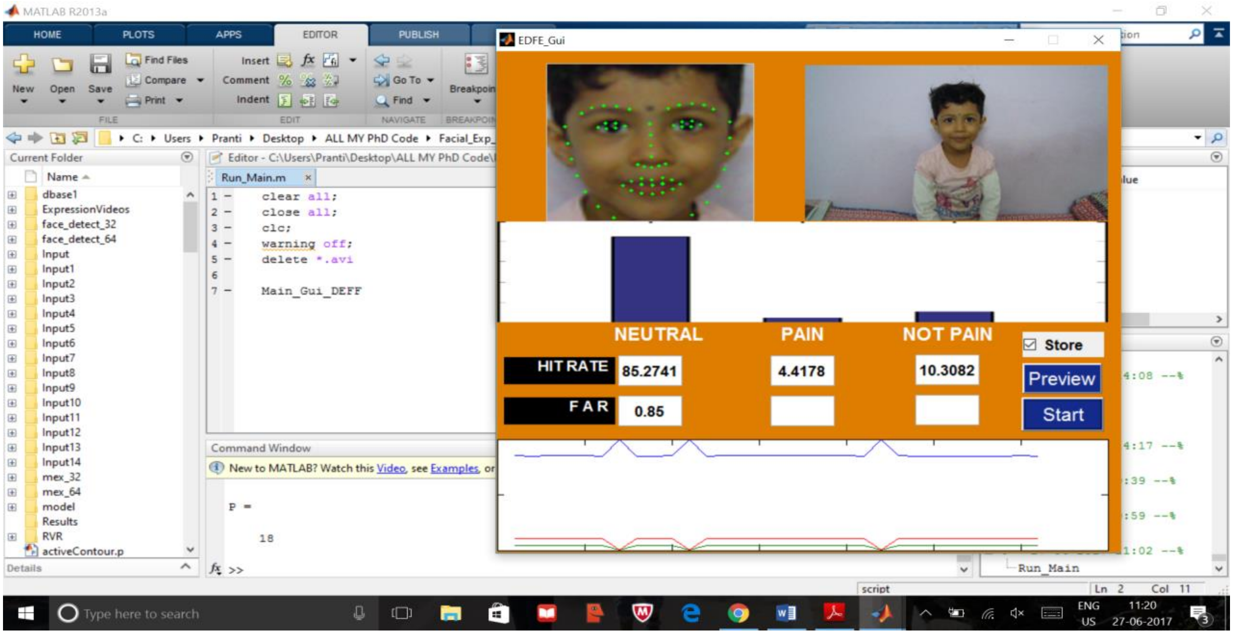

Figure ZZ: Testing part of a sample video using Multilayer Back Propagation Neural Network (neutral_face)
